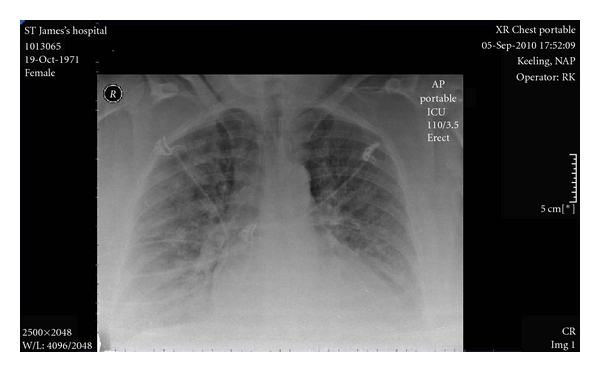# Dyspnoea at Term in an Obese Mother

**DOI:** 10.1155/2011/317376

**Published:** 2011-09-22

**Authors:** Vicky O'Dwyer, Yvonne O'Brien, Nadine Farah, Michael J. Turner

**Affiliations:** UCD Centre for Human Reproduction, Coombe Women and Infants University Hospital, Dublin 8, Ireland

## Abstract

Peripartum cardiomyopathy is a serious, potentially life-threatening heart disease of uncertain aetiology in previously healthy women. We report a morbidly obese woman who presented with peripartum shortness of breath. We discuss the differential diagnosis of dyspnoea in pregnancy and highlight the complexity of care of the morbidly obese woman.

## 1. Introduction

Maternal obesity is associated with an increased risk of complications for the mother and her offspring during pregnancy, at delivery and during the postpartum period [1, 2]. Although dyspnoea is a common symptom in obese pregnant women, other differential diagnoses, however, should not be overlooked.

Peripartum cardiomyopathy (PPCM), for example, is a rare and potentially life-threatening differential diagnosis. This report highlights the complexity of care which may be required for a morbidly obese pregnant woman.

## 2. Case Report

A 35-year-old European first-time mother with an unplanned pregnancy presented late for antenatal care at 35 weeks gestation. In her past medical history, she had asthma for which she was using a salbutamol inhaler. At presentation her weight was 147 kg and her Body Mass Index (BMI) was 54 kg/m^2^. An oral glucose tolerance test was normal. She was seen regularly in the antenatal clinic where she was found to be normotensive with no proteinuria. At her visits she, however, did complain of dyspnoea on exertion, bilateral leg swelling, and excessive weight gain especially in the third trimester. However, these findings were attributed to her pregnancy.

At 40 weeks and three days, she presented with bilateral leg oedema, erythema, and tenderness. She was admitted and had a Doppler ultrasound performed which ruled out a deep venous thrombosis. She was treated with antibiotics and had physiotherapy. An ultrasound scan was performed to assess fetal well-being which estimated the fetal weight to be 4755 g and demonstrated polyhydramnios. At 41 weeks and one day, the patient had prelabour rupture of her membranes and was induced after 24 hours with an oxytocin infusion.

On the delivery suite, it was difficult to monitor the fetal heart rate by an external monitor and a fetal scalp electrode was applied. The patient was contracting efficiently on the oxytocin. An epidural was sited, which made it difficult to palpate the contractions. There was no change in the cervical dilation after being six hours on the oxytocin infusion and an emergency caesarean section was carried out. 

There was abdominal subcutaneous oedema and ascites noted at the caesarean section. Although the procedure was technically difficult due to maternal size, there were no complications. A healthy male was delivered weighing 4750 g. Prophylactic antibiotics and low molecular weight heparin were prescribed postoperatively.

On day four, the woman complained of chest tightness and dyspnoea. On examination, she was noted to have a tachycardia. She was normotensive and apyrexial. Her oxygen saturation was 97% on room air and an arterial blood gas was normal. On auscultation of her lungs, there was a bilateral wheeze noted and her heart rate was regular with an S3 gallop. An electrocardiogram showed a normal sinus rhythm. A computed tomography was performed which demonstrated mild inflammatory changes and ruled out a pulmonary embolism. She was commenced on antibiotics, nebulisers, and intravenous hydrocortisone to treat an exacerbation of asthma, possibly due to a chest infection. The following day her dyspnoea increased with no change in her clinical examination. A chest X-ray performed demonstrated cardiomegaly with increased vascular congestion bilaterally (Figure 1). An arterial blood gas on room air demonstrated hypoxia.

She was transferred to the intensive care unit where a transthoracic echocardiogram demonstrated a globally hypokinetic left ventricle, an ejection fraction of less than 30%, and mild/moderate tricuspid regurgitation. The working diagnosis at this point was peripartum cardiomyopathy with a superimposed respiratory tract infection. She was given frusemide and a glyceryl trinitrate infusion for the management of her pulmonary oedema and fluid overload. The antibiotics were continued for the suspected superimposed infection.

After the initial treatment, she was commenced on an ACE inhibitor and beta-blocker for the long-term management of cardiomyopathy and was discharged home on day 14 postpartum. An echocardiogram at five months postpartum showed a left ventricular EF of 50% and the ACE inhibitor and a Beta Blocker were continued. At six months postpartum, a Mirena coil was inserted for contraceptive purposes.

## 3. Discussion

Obesity is an increasing problem and presents one of the greatest challenges to the practising clinician, across all disciplines. The incidence of obesity has increased dramatically over the past twenty years. The prevalence of adult obesity exceeds 15% in most countries, 20% in Ireland and the rest of Europe, and more than 30% in the United States of America [2].

Maternal obesity is associated with an increase in medical complications such as gestational diabetes mellitus, pre-eclampsia/hypertension, venous thromboembolism, and infection. Women who are obese have an increased rate of obstetric interventions such as induction of labour, operative vaginal delivery, and caesarean section. Women who are obese are more likely to have pregnancies with fetal complications such as congenital malformations, unexplained stillbirth, fetal macrosomia, and dizygotic twins. After delivery, obese mothers have an increased rate of postpartum haemorrhage and of difficulties with breastfeeding [1, 2]. 

Delivering care to obese women is challenging, particularly in cases of morbid obesity (BMI > 39.9 kg/m^2^). Excessive maternal adiposity hinders antenatal fetal assessment by ultrasound, hinders intravenous access and epidural anaesthesia, and hinders operative exposure when surgical intervention is necessary. Our case also highlights that although dyspnoea is a common symptom in obese pregnant women, other differential diagnoses need, however, not be overlooked.

PPCM is a rare but potentially life-threatening disorder of unknown aetiology and pathophysiology. The incidence of PPCM varies worldwide [3]. Reported incidence of PPCM in non-African countries ranges between 1 : 3,000 to 1 : 15,000 live births [4, 5]. The diagnosis of PPCM is based on four primary diagnostic criteria, and these are (1) the development of the disease in the last month of pregnancy or within 5 months of delivery, (2) absence of an identifiable cause of heart failure, (3) absence of recognisable heart disease before the last month of pregnancy, and (4) left ventricular systolic dysfunction demonstrated by classical echocardiographic criteria [6].

The strongest risk factor for PPCM appears to be African-American ethnicity [7]. Other reported risk factors include age, pregnancy-induced hypertension or preeclampsia, multiparty, multiple gestations, obesity, chronic hypertension, and prolonged use of tocolytics [8, 9]. Symptoms of PPCM include fatigue, oedema, and dyspnoea [10]. Other unusual presentations include multiple thrombotic events and acute hypoxia [11, 12]. Signs can include tachycardia, pulmonary rales, and an S3 heart sound [13]. Such signs and symptoms overlap with those of many other conditions, ranging from normal pregnancy to pulmonary emboli and upper respiratory infection.

ECG abnormalities are often noted on presentation, most commonly sinus tachycardia, nonspecific ST segment changes, LV hypertrophy, premature ventricular contraction, and bundle branch block [14]. However, the ECG may show no changes [15]. Chest radiographs can show signs of pulmonary congestion and cardiac enlargement and even pleural effusions in some cases [16]. Echocardiograms usually show decreased contractility and LV enlargement without hypertrophy [5].

The treatment of PPCM is the same as for other forms of congestive heart failure, except angiotensin converting enzyme inhibitors and angiotensin receptor blockers, which are contraindicated in pregnancy [17]. Patients with PPCM are also at high risk of thrombus formation and anticoagulation should be considered [18]. The best time to discontinue these medications is unknown but their use should be continued for at least one year [19]. If medical treatment is not successful, heart transplantation may be the last resort.

About half the women are expected to fully recover cardiac function [17]. Regardless of recovery, however, a second pregnancy is usually not recommended as PPCM recurs in more than 30% of subsequent pregnancies which puts mother and baby at great risk [17].

Our case highlights the potential complexity of managing morbidly obese pregnant women. Although there may be a focus on common complications associated with obesity, one needs to be vigilant for the less common complications such as PPCM.

## Figures and Tables

**Figure 1:**